# Flavonoid Composition and Bioactivities of *Nymphaea* ‘Blue Bird’: Analysis, Purification, and Evaluation

**DOI:** 10.3390/life15121895

**Published:** 2025-12-11

**Authors:** Mengjun Zhou, Enbo Wang, Xin Li, Xia Zhao, Jianan Xu, Wentao Wu, Ying Zhao

**Affiliations:** 1 Key Laboratory of Ministry of Education for Genetics and Germplasm Innovation of Tropical Special Trees and Ornamental Plants, Hainan Biological Key Laboratory for Germplasm Resources of Tropical Special Ornamental Plants, College of Tropical Agriculture and Forestry, Hainan University, Haikou 570228, China; baroque2014@outlook.com (E.W.); lx19116968146@163.com (X.L.); zhaopray@163.com (X.Z.); xujianan9343@163.com (J.X.); 2 Hainan Forestry Group Co., Ltd., Haikou 570228, China; mengjunzhou@126.com

**Keywords:** *Nymphaea*, flavonoids, biological activity, metabolomics

## Abstract

*Nymphaea* ‘Blue Bird’, a tropical water lily prized for its ornamental appeal, has been less explored as a source of bioactive flavonoids. This study developed an efficient extraction and purification protocol for flavonoids from this plant and compared their distribution and bioactivities across different tissues. Supercritical CO_2_ fluid extraction (SFE) proved optimal, yielding 2.56% under conditions of 24.3 MPa, 39 °C, 91 min, and a CO_2_ flow rate of 16 L/min. Subsequent purification with HPD500 macroporous resin enhanced flavonoid purity from 3.05% to 11.46%. Among the tissues analyzed, petals contained the highest levels of total flavonoids (6.43 mg/g) and total phenolics (45.71 mg/g), and exhibited the most potent antioxidant (as shown by the lowest EC_50_ values for ABTS^+^ and DPPH scavenging) and broad-spectrum antibacterial activities (indicated by the lowest MIC and MBC against *Staphylococcus aureus*, *Pseudomonas aeruginosa*, *Escherichia coli*, and *Candida albicans*). Antibacterial efficacy was generally superior against Gram-positive bacteria. Widely targeted metabolomics identified 560 metabolites, predominantly flavonols and flavonoids. Principal component and cluster analyses revealed tissue-specific metabolite profiles. KEGG enrichment analysis underscored the significance of the flavonoid biosynthetic pathway, and key differential metabolites—such as luteolin, myricetin, taxifolin, and quercetin—were strongly correlated with the observed bioactivities. These results highlight *N*. ‘Blue Bird’ petals as a promising source of natural antioxidants and antimicrobials, providing a scientific basis for their future functional applications.

## 1. Introduction

In recent years, flavonoids have gradually attracted social attention due to their functions such as anti-aging, antioxidant, antibacterial, and inhibition of tumor cells, and have been widely applied in industries such as food, pharmaceuticals, and cosmetics [1,2]. Research has found that flavonoids mainly accumulate in parts such as petals, leaves, and stamens of *Nymphaea* plants [3,4]. To date, the research on *Nymphaea* plants has mostly focused on petals, such as the mechanism between flower color and flavonoids, and the antioxidant and anti-inflammatory effects of flavonoid extracts from petals. A total of 117 flavonoids were detected in the flower pigments of water lily petals, including 20 anthocyanins, 9 chalcone glycosides, 2 flavanones, 3 flavanone glycosides, 4 flavones, 5 flavone glycosides, 6 flavonols and 68 flavonol glycosides [5]. The composition of flavonoids is closely related to flower color and tissue location. In blue-colored varieties such as *Nymphaea* ‘Blue Bird’, the anthocyanins are mainly delphinidin B-ring 3′-position glycosylated derivatives, with a small amount of delphinidin C-ring 3-position glycosylated derivatives [6]. Research on water lily flowers has found that the dominant metabolite epicatechin in the pistils of water lilies is formed through the biosynthesis pathway of phenylpropanoid compounds, the dominant metabolites naringenin, kaempferol and cyanidin in the petals are synthesized through the flavonoid biosynthesis pathway, the dominant metabolite rutin in the stamens is synthesized through the biosynthesis pathways of flavones and flavonols, and aspartic acid, tyrosine and glutamic acid are the dominant metabolites in the pistils of water lilies, with their contents higher than those of other tissues of water lily flowers [7]. However, there is still a lack of research on flavonoids in tropical water lilies. Especially regarding the efficient extraction and purification process of flavonoids in the *Nymphaea* ‘Blue Bird’ variety, the systematic comparison of flavonoid metabolic profiles in different tissue parts, and the correlation mechanism between its metabolic profile and antioxidant and antibacterial functions, there have been no reports yet.

Tropical water lilies are a general term for species in the genus *Nymphaea* of the family *Nymphaea*ceae that are widely distributed in tropical regions. In particular, the *Nymphaea* ‘Blue Bird’ among tropical water lily species has a long flowering period, high ornamental value, and great economic development potential. Elegami et al. studied the metabolome of the petals of *Nymphaea* ‘Blue Bird’ during the opening and closing stages. The results showed that a total of 455 metabolites were identified in the petals, among which 100 were flavonoids. In the open petals, 12 different flavonoids were identified, including 2 chalcones, 3 flavanols, 5 dihydroflavones, and 2 dihydroflavonols [8].

Currently, research on the component analysis and activity of secondary metabolites in tropical water lilies is still relatively scarce. As polyphenolic secondary metabolites widely present in plants, flavonoids possess biological activities and pharmacological effects such as antibacterial, anti-inflammatory, antioxidant, and anti-tumor properties. In this study, *Nymphaea* ‘Blue Bird’ was taken as the research object. Optimize the process flow of total flavonoid extraction and purification through supercritical CO_2_ fluid extraction and column chromatography, respectively. Flavonoids were identified from the extraction of different parts of *Nymphaea* ‘Blue Bird’ using high-performance liquid chromatography. Five types of macroporous resins were selected for the purification of flavonoids from *Nymphaea* ‘Blue Bird’, and purified products of *N.* ‘Blue Bird’ flavonoids were obtained. In addition, the antibacterial activity of *Nymphaea* extracts from different parts against four pathogenic bacteria was evaluated by measuring the diameter of the antibacterial zone, minimum inhibitory concentration, and minimum bactericidal concentration. Furthermore, relevant network analysis was used to screen the central metabolic components related to their ability to resist the four pathogenic bacteria. In addition, targeted metabolomics was used to detect and analyze flavonoids in different parts of the water lily, and differential metabolites were screened. K-means was combined to analyze the synthetic pathways involved in the differential metabolites, exploring the metabolites of flavonoids in *Nymphaea* ‘Blue Bird’ and the biological activities of flavones, so as to provide a basis for the screening of high-quality resources of tropical water lilies, genetic improvement, and the comprehensive utilization and development of flavonoid metabolites.

However, there is still a lack of systematic research on the extraction and purification process, tissue distribution characteristics, and their association with antioxidant and antibacterial activities of flavonoids in the tropical water lily variety *Nymphaea* ‘Blue Bird’. Therefore, the aim of this study is to: (1) establish and optimize the supercritical CO_2_ extraction and resin purification process of ‘Bluebird’ flavonoids; (2) Compare the differences in flavonoid content, antioxidant and antibacterial activity among different tissues; (3) Using extensive targeted metabolomics to analyze its flavonoid metabolism profile, screening key differential metabolites related to biological activity, and providing scientific basis for its further development and utilization.

## 2. Materials and Methods

### 2.1. Experimental Materials

The material used in this experiment was *Nymphaea* ‘Blue Bird’, which was sourced from the water lily germplasm resource nursery of the College of Tropical Agriculture and Forestry, Hainan University, located in Haikou City, Hainan Province. In August 2023, six parts including petals, stamens, stigmas, flower stems, leaves, and roots were collected from healthy adult *Nymphaea* ‘Blue Bird’ plants (Supplementary Table S1). Upon collection, samples were immediately snap-frozen in liquid nitrogen and stored at −80 °C prior to flavonoid metabolite profiling, with all data derived from three biological replicates.

Rutin standard (HPLC ≥ 98%), gallic acid, L-ascorbic acid, ferric chloride hexahydrate, ferrous sulfate (FeSO_4_·7H_2_O), hydrochloric acid, sodium nitrite, aluminum nitrate, sodium carbonate, absolute ethanol are all of analytical grade, purchased from Beijing Solarbio Technology Co., Ltd. (Beijing, China).

*Escherichia coli* (ATCC 25922), *Staphylococcus aureus* (ATCC 27217), *Pseudomonas aeruginosa* (ATCC 9027), and *Candida albicans* (ATCC 10231) were stored at −80 °C in Luria–Bertani (LB) broth and Sabouraud broth, respectively, each containing 25% glycerol. Prior to each experiment, the test strains were cultured in the specified broths at 37 °C with shaking for 24–48 h.

### 2.2. Overview of Analytical and Purification Methods

The detailed procedures for the determination of total flavonoid content, single-factor and response surface methodology experiments, macroporous resin screening, and adsorption/desorption studies are provided in the Supplementary Methods file for clarity and conciseness. These comprehensive protocols include the rutin standard curve method, optimization of supercritical CO_2_ fluid extraction parameters, screening and kinetic studies of five macroporous resins, and the systematic investigation of factors affecting resin adsorption and desorption. All extraction and purification experiments were independently repeated three or more times (*n* ≥ 3).

### 2.3. Determination of Minimum Inhibitory Concentration and Minimum Bactericidal Concentration

The flavonoid extract was dissolved in anhydrous ethanol, evaporated to dryness under reduced pressure at 40 °C, and obtained as a dry powder. Prior to use, the powder was resuspended in sterile water to the desired concentration. Solvent control wells containing an equal amount of anhydrous ethanol subjected to the same evaporation and resuspension process were established. MIC was determined using the two-fold broth microdilution method in 96-well plates. For the procedure, 0.2 mL of a 40 mg/mL water lily flavonoid solution was added to the first well of the plate. Wells 2 to 11 each received 0.1 mL of the micro-broth medium, while well 12 received 0.2 mL of the broth medium alone. Then, 0.1 mL of the flavonoid solution was transferred from the first well to the second well, mixed by aspirating and pipetting 5 times. This two-fold serial dilution process was repeated sequentially up to the 10th well, from which 0.1 mL was discarded after mixing. Subsequently, 0.1 mL of a bacterial suspension at a concentration of 10^−5^ to 10^−6^ CFU/mL was added to wells 1 through 11. Well 11 served as the “positive control” (bacterial growth control), and well 12 as the “medium blank control”. Finally, the bacterial plates were incubated at 37 °C for 24–48 h, and the *C. albicans* plates were incubated at 28 °C for 48–72 h. The MIC was defined as the concentration in the last clear well showing no visible growth.

For MBC determination, 0.1 mL aliquots from the well identified as the MIC, as well as from the two preceding wells (with higher concentrations), were sampled. For bacteria, these aliquots were spread on LB agar plates and incubated at 37 °C for 24–48 h. For *C*. *albicans*, the aliquots were spread on Sabouraud dextrose agar plates and incubated at 28 °C for 48–72 h. The MBC was defined as the lowest concentration among these three where fewer than 5 colonies grew on the agar plate.

### 2.4. Determination of Inhibition Zone

The inhibition zone of water lily flavonoids was determined using the punch-hole diffusion method [9]. Briefly, 0.1 mL of bacterial suspensions (*E*. *coli*, *S*. *aureus*, and *P*. *aeruginosa*) at concentrations of 10^−5^ to 10^−6^ CFU/mL were spread evenly onto LB agar plates, respectively. Similarly, a *C*. *albicans* suspension was spread onto Sabouraud dextrose agar plates. Then, solutions of water lily flavonoids at concentrations of 600, 300, and 150 mg/mL were pipetted into the corresponding wells on the plates inoculated with the test strains. After incubation under the conditions mentioned previously, the antibacterial effects were observed. Vitamin C (100 mg/mL) and fluconazole (100 mg/mL) were used as positive controls for bacteria and fungi, respectively. A 100 mg/mL Tween-80 solution served as the negative control, and sterile water was used as the blank control. The experiment was performed in triplicate. The diameter of the inhibition zones for each plate was measured using the cross-cross method, and the average value was calculated. The inhibition rate was subsequently determined [10].

### 2.5. Metabolite Extraction Procedure

Prior to analysis, samples were freeze-dried, pulverized (30 Hz, 15 min), and then 50 mg of the powder was extracted with 1200 μL of −20 °C 70% aqueous methanol (with internal standard). The extraction involved repeated vortexing (6 cycles of 30 s every 30 min). After centrifugation (12,000 rpm, 3 min), the supernatant was filtered through a microporous membrane for subsequent use.

### 2.6. Chromatography and Mass Spectrometry Acquisition Conditions

Liquid Chromatography Conditions: Separation was achieved using an Agilent SB-C18 column (Agilent Technologies Inc., Santa Clara, CA, USA) (100 mm × 2.1 mm, 1.8 µm). The mobile phase comprised (A) ultrapure water with 0.1% formic acid and (B) acetonitrile with 0.1% formic acid. A linear gradient was applied as follows: 5% B at 0 min, increasing to 95% B at 9 min, held isocratic until 10 min, then returned to 5% B at 11.1 min, with re-equilibration at 5% B until 14 min. The flow rate, column temperature, and injection volume were set at 0.35 mL/min, 40 °C, and 2 µL, respectively.

Mass Spectrometric Parameters: The ESI source temperature was maintained at 550 °C with ion spray voltages of 5500 V and −4500 V. The nebulizer (Gas I), heater (Gas II), and curtain gas flows were 50, 60, and 25 psi, respectively; collision energy was applied at a “high” setting. Analysis was conducted in MRM mode using medium collision gas. Following compound-specific optimization of the declustering potential (DP) and collision energy (CE), a scheduled MRM method was defined to monitor the unique transitions for each metabolite at its predetermined retention time. The sample extraction and LC-MS/MS injection sequence were randomized to eliminate potential batch effects.

### 2.7. Qualitative and Quantitative Analysis of Metabolites

The number of metabolites in *Nymphaea* ‘Blue Bird’ was subjected to Log 2 transformation and normalization for general statistical analysis. Using R software 4.0.4, the accumulation patterns of metabolites in 18 samples were analyzed through hierarchical cluster analysis (HCA), PCA, and OPLS-DA. When the FC of a metabolite was ≥2 or ≤0.5, it was considered to have a significant difference. Finally, the KEGG database with a *p*-value < 0.05 or VIP > 1 was selected to study the metabolites in different tissue parts of *Nymphaea* ‘Blue Bird’. Metabolite identification was based on mass spectrometry matching with standards, secondary mass spectrometry data alignment, and searches in internal/public databases (e.g., MetWare, KEGG), with confidence levels at Level 2 or higher.

### 2.8. Data Analysis and Mapping

Excel and IBM SPSS Statistics 26 software were used for data statistical analysis, and Origin 2021 software was used for mapping. Bioinformatics analysis was performed in R. One-way ANOVA with Duncan’s test was used for all relevant comparisons. After log2 transformation and unit variance scaling, unsupervised PCA (prcomp) and supervised OPLS-DA (MetaboAnalystR package OPLSR.Anal function of R language 4.0.4 software) were utilized. The OPLS-DA model, validated by 200 permutations, provided VIP scores for selecting significant metabolites (VIP ≥ 1 and |Log2FC| ≥ 1). HCA visualized data structure via a heatmap package. Metabolites were annotated in KEGG Compound and mapped to pathways, with enrichment significance (*p*-value) computed via a hypergeometric test.

## 3. Results

### 3.1. Optimization of Flavonoid Extraction and Purification

To establish an efficient and green method for flavonoid recovery, SFE was employed. To determine the optimum extraction conditions for pressure, temperature, time, and CO_2_ flow rate, we employed a Box–Behnken experimental design, building upon insights from initial single-factor tests. The specific design layout and response data are provided in Supplementary Table S2.

The data were fitted to a quadratic model, which was found to be highly significant (*p* < 0.0001). The interaction between pressure and flow rate was among the most significant factors affecting the yield. The model predicted an optimal extraction yield of 2.16% under the conditions of 24.32 MPa, 38.99 °C, 91.24 min, and a flow rate of 16.02 L/min. For practical operation, parameters were adjusted to 24.3 MPa, 39 °C, 91 min, and 16 L/min. Validation experiments under these conditions yielded an average flavonoid extraction rate of 2.56 ± 0.11%, confirming the model’s robustness.

**Figure 1:**
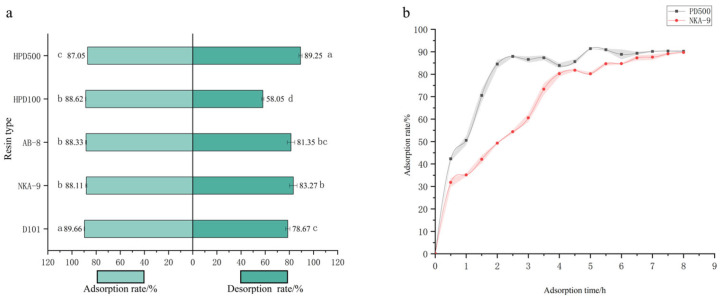
Screening of five macroporous resins. Note: (**a**) Adsorption and resolution of five macroporous resins; (**b**) Static adsorption curves of two macroporous resins. Different letters above bars indicate significant differences (*p* < 0.05).

For purification, five macroporous resins (HPD500, HPD100, AB-8, NKA-9, D101) were evaluated (Figure 1a,b). While adsorption rates were similar (~87–89%), HPD500 exhibited the highest desorption rate (89.25%). Static adsorption kinetics revealed that the process for both HPD500 and NKA-9 fitted the pseudo-second-order model better (R^2^ > 0.97), with HPD500 reaching equilibrium faster (2 h vs. 4 h). Consequently, HPD500 was selected for further optimization. The optimal purification conditions were determined as follows: The sample size is 5 mL (material to liquid ratio 10:1 mL/g), pH 3.0, sample concentration of 0.359 mg/mL; elution with 30 mL of 90% ethanol at a flow rate of 1.5 mL/min (Supplementary Figure S1). This process successfully increased the purity of the total flavonoids from 3.05% in the crude extract to 11.46% in the purified product, representing a 3.76-fold enrichment.

### 3.2. Variation in Bioactive Components and Antioxidant Activities

**Figure 2:**
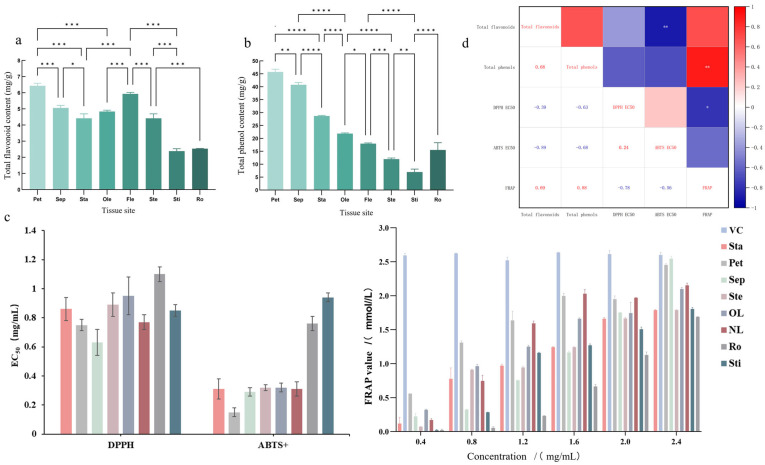
Content of total flavonoids (**a**), total phenolics (**b**), antioxidant activity ((**c**) EC_50_ and FRAP values), and correlation coefficients between content and antioxidant indicators (**d**) in different parts of *N.* ‘Blue Bird’. Note: Pet: Petal, Sep: Sepal, Sta: Stamen, OL: Old Leaf, NL: New Leaf, Ste: Stem, Ro: Root, Sti: Stigma. The asterisks (*, **, ***, ****) above the columns indicate significant differences at *p* < 0.05, *p* < 0.01, *p* < 0.001, and *p* < 0.0001, respectively.

The contents of total flavonoids and total phenolics varied significantly across different parts of *N.* ‘Blue Bird’ (Figure 2a,b). Petals possessed the highest levels of both total flavonoids (6.43 mg/g) and total phenolics (45.71 mg/g), followed by sepals and new leaves. The stigma and root showed the lowest contents. This distribution suggests that petals are the primary accumulation site for these bioactive compounds.

The antioxidant activities of the flavonoid extracts from different parts were evaluated using DPPH and ABTS^+^ radical scavenging assays and the Ferric reducing antioxidant power (FRAP) test (Figure 2c). Consistent with the content analysis, petals and sepals exhibited the strongest antioxidant capacity, as evidenced by their low EC_50_ values for radical scavenging and high FRAP values.

Correlation analysis revealed a strong negative correlation between total flavonoid content and ABTS^+^ EC_50_ value (R = −0.894), and a strong positive correlation between total phenolic content and FRAP value (R = 0.883) (Figure 2d). This indicates that the antioxidant potency is largely dependent on the concentration of these compounds, although the specific composition of flavonoids also plays a critical role.

### 3.3. Assessment of Antibacterial Activities

**Figure 3:**
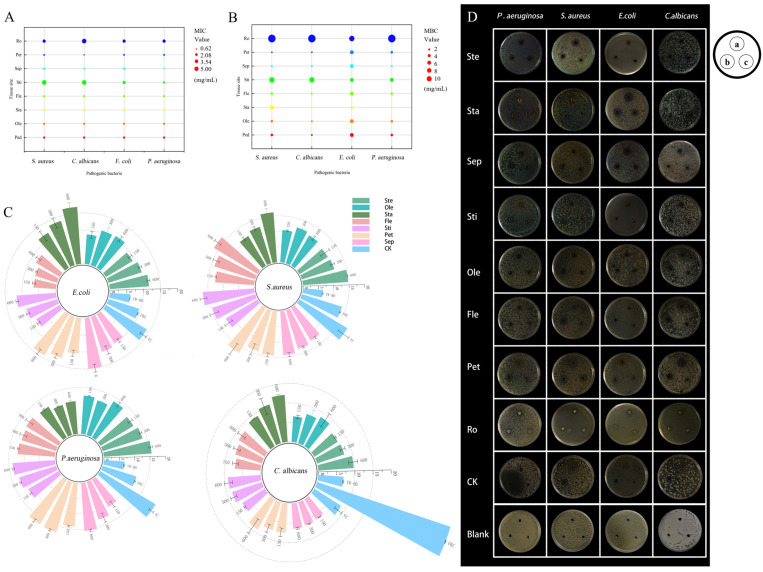
Evaluation of antibacterial activity of flavonoids extracted from *N.* ‘Blue Bird’. Note: MIC (**A**) and MBC (**B**) values of flavonoid extracts from different parts against foodborne pathogens. (**C**) Antimicrobial ring diameters (mm) at different tissue sites. (**D**) Antibacterial effects of flavonoids from various tissue parts on four foodborne pathogens. Experimental group a, b, c were 600 mg/mL, 300 mg/mL, 150 mg/mL, respectively; CK group a, b, c were VC, fluconazole, Tween-80, respectively.

The antibacterial and antifungal properties of the flavonoid extracts were assessed against two Gram-positive bacteria (*S. aureus*), two Gram-negative bacteria (*E. coli* and *P. aeruginosa*), and the fungal pathogen *C. albicans*. This evaluation was conducted by employing the standard metrics of Minimum Inhibitory Concentration (MIC) and Minimum Bactericidal Concentration (MBC), as presented in Figure 3A,B.

Extracts from petals and sepals exhibited dominant efficacy against all tested pathogens, showing the lowest MIC and MBC. With the notable exception of stamen flavonoids being distinctly more effective against Gram-negative bacteria, inhibitory activity was generally stronger against Gram-positive *S. aureus* than against Gram-negative *E. coli* and *P. aeruginosa*. This selectivity is attributed to the structural complexity of the Gram-negative outer membrane, which significantly reduces compound permeability [3]. The agar well diffusion assay further corroborated these findings, with petal extracts producing the largest inhibition zones against *S. aureus* and *P. aeruginosa* (Figure 3C,D).

### 3.4. Qualitative and Quantitative Analysis of Flavonoid Metabolites

**Figure 4:**
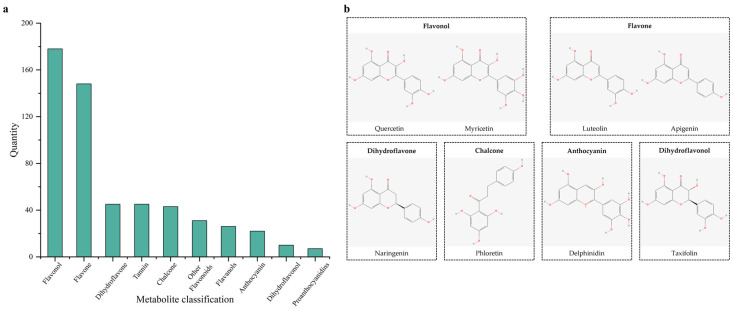
Distribution of identified flavonoid metabolites and chemical structures of representative compounds in *N.* ‘Blue Bird’. Note: (**a**) Distribution of identified flavonoid metabolites in *N.* ‘Blue Bird’. (**b**) Chemical structure diagram of representative metabolites.

Through the detection and analysis of flavonoid metabolites, 560 metabolites of *Nymphaea* ‘Blue Bird’ were obtained. Among them, 515 flavonoid compounds accounted for 90.71% of all metabolites, and 45 tannin compounds accounted for 9.29% of all metabolites (Figure 4). Among the flavonoid metabolite products, in descending order, there were 178 flavonols, 148 flavones, 45 dihydroflavones, 43 chalcones, 31 other flavonoids, 26 flavanols, 22 anthocyanins, 10 dihydroflavonols, 7 proanthocyanidins, and 5 aurones, a total of 10 types of substances. Flavonols were mainly derivatives of quercetin, kaempferol, myricetin, and isorhamnetin. Quercetin can reduce the content and activity of *P. aeruginosa* elastase *B. flavones* were mainly composed of tricin and apigenin, also including luteolin, chrysoeriol, and other derivatives. Naringenin can be catalyzed by flavone and flavonol reductase to form apigenin derivatives, and apigenin is reduced to naringenin by reductase in the human intestine and then absorbed by the human body. Dihydroflavones mainly had eriodictyol and naringenin as the main metabolites, also including hesperetin and pinocembrin. Chalcones were mainly phloretin and its derivatives. Epigallocatechin derivatives were abundant in flavanols, and they were the starting monomers for the synthesis of plant polymeric anthocyanins or condensed tannins. Dihydroflavonols included metabolites such as taxifolin and aromadendrin. In anthocyanins, delphinidin and cyanidin and their derivatives were abundant. In aurones, typical ones included heliosupine and aurovertin.

### 3.5. Principal Component Analysis of Overall Samples and Sample Correlation Analysis

**Figure 5:**
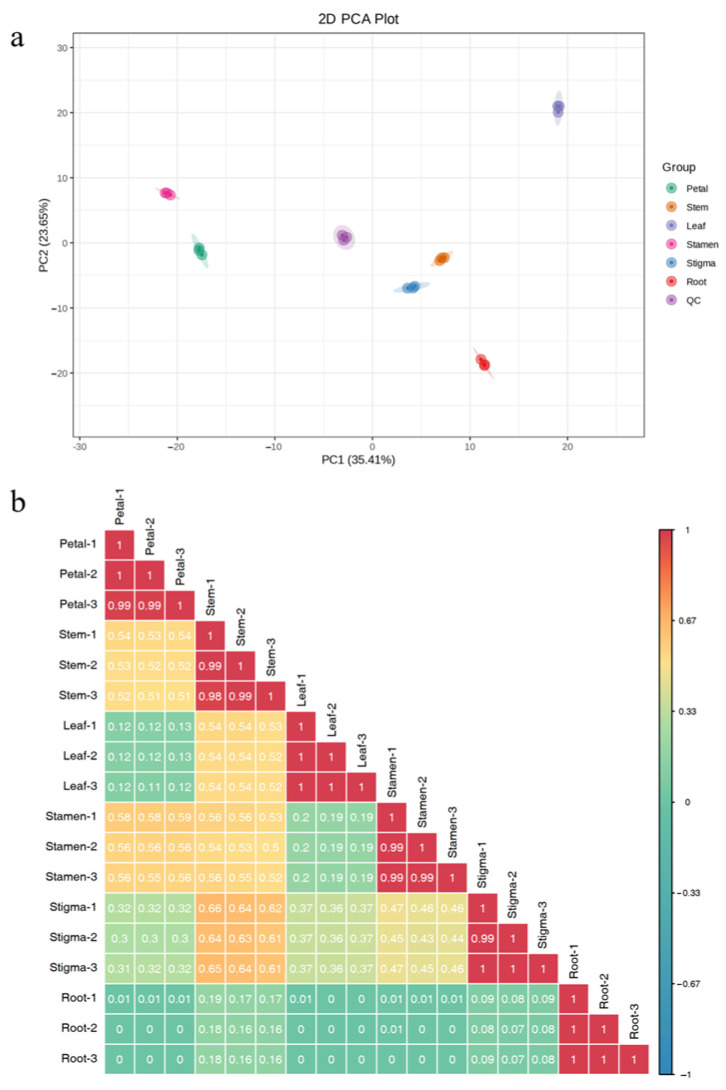
Principal component analysis of overall samples and sample correlation analysis. Note: (**a**) PCA results of each part of *Nymphaea* ‘Blue Bird’. (**b**) Correlation analysis chart between samples.

Following PCA of the 515 metabolites detected in six parts of *Nymphaea* ‘Blue Bird’, the first and second principal components were found to represent 35.41% and 23.65% of the total variance, respectively. The first principal component was much higher than the second principal component (Figure 5a). From the results, the three samples of each part independently aggregated, respectively, and each part also showed an obvious separation trend, indicating that there were compounds with significant differences in each part. Especially in the leaf, stamen, and petal parts, they could be used as markers to identify the differential metabolites of different parts of *Nymphaea* ‘Blue Bird’. The flower stem and stigma parts of *Nymphaea* ‘Blue Bird’ were relatively close, suggesting that the metabolite compositions of these two parts were relatively similar. According to the characteristics of the correlation coefficient, the biological replicates among samples could be indicated. The correlation coefficients of biological replicates within each part were all greater than 0.9, indicating a strong correlation among them. The low correlation coefficients between groups indicated that there were certain differences in metabolites between groups (Figure 5b).

### 3.6. Hierarchical Cluster Analysis

**Figure 6:**
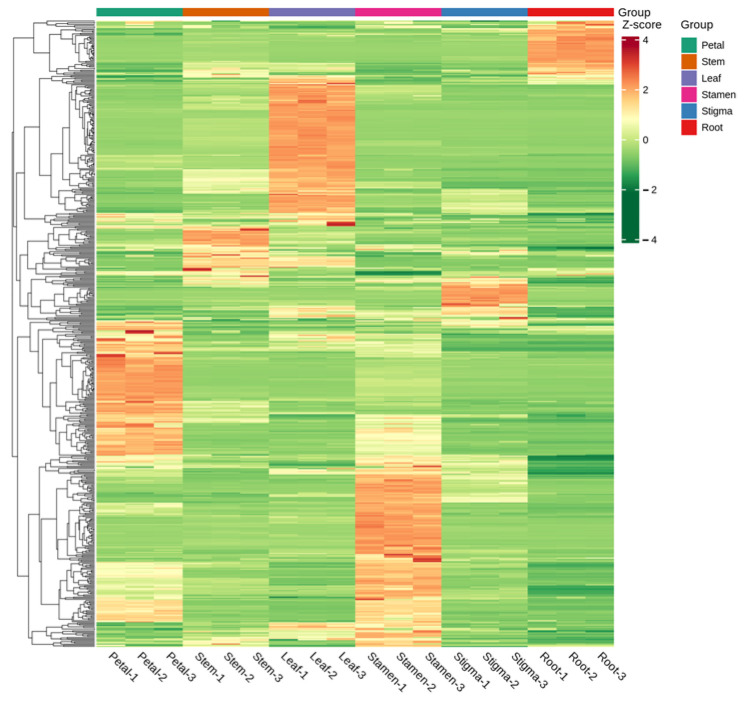
Cluster heat map of each group of samples.

The differences in the accumulation patterns of flavonoid metabolites in various parts can be analyzed through a cluster heatmap. There were obvious differences in flavonoids among different parts. Overall, the heatmap could be divided into 5 clusters. The metabolites in Cluster 1 had the highest content in the roots, while the flavonoid metabolites in Cluster 2 had the highest content in the leaves. Cluster 3 had a higher content in the flower stems and stigmas and a lower content in the roots. Cluster 4 had the highest content in the petals and a lower content in the roots. Cluster 5 had the highest content in the stamens, and the lowest content was still in the roots (Figure 6). There were significant differences in flavonoid metabolites among different parts of *Nymphaea* ‘Blue Bird’. The metabolite content accumulated more in the stamens, petals, and leaves. Meanwhile, the cluster heatmap analysis could also indicate the good homogeneity of biological replicates.

### 3.7. Analysis of Differential Metabolites

**Figure 7:**
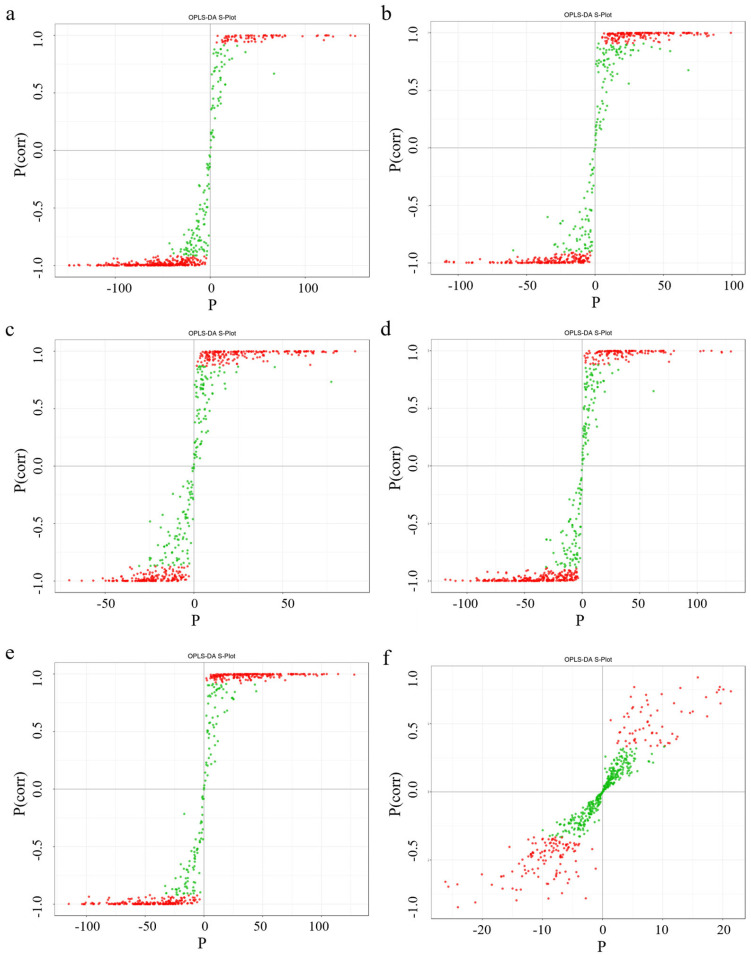
Selected orthogonal partial least squares discriminant analysis S-plot. Note: The abscissa corresponds to the covariance between principal components and metabolites, while the ordinate represents their correlation coefficient. Metabolites positioned in the upper-right and lower-left corners exhibit more pronounced differential expression. In this plot, red and green dots denote metabolites with VIP values > 1 and ≤1, respectively. From left to right, the picture cascade is Sti-Pet, Ste-Pet, Sta-Pet, Ro-Pet, Lea-Pet, Pet-Ste-Lea-Sta-Sti-Ro. (**a**) Sti-Pet; (**b**) Ste-Pet; (**c**) Sta-Pet; (**d**) Ro-Pet; (**e**) Lea-Pet; (**f**) Pet-Ste-Lea-Sta-Sti-Ro.

**Figure 8:**
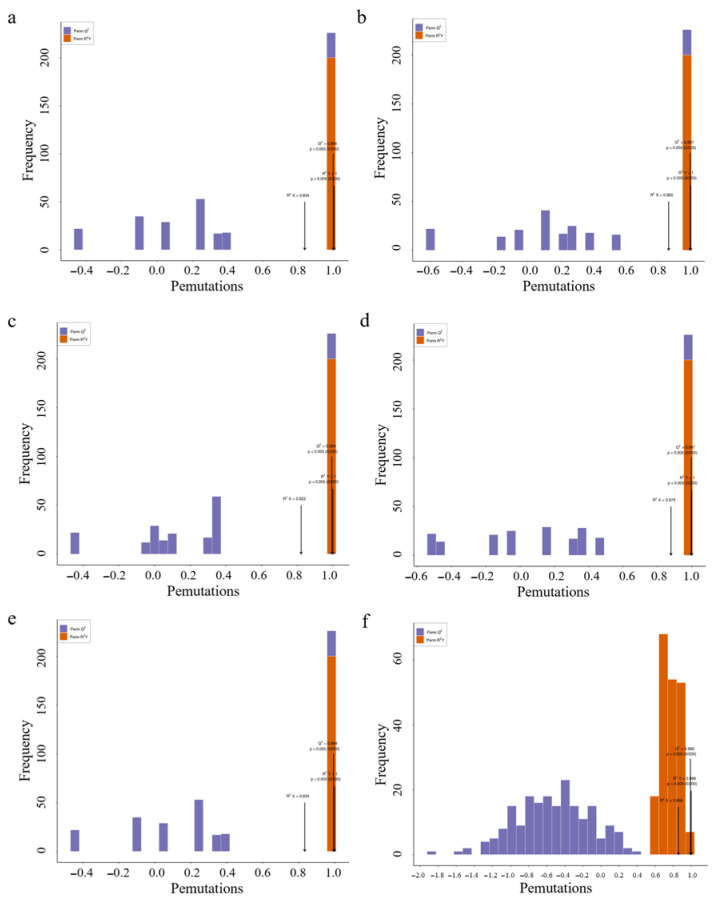
Selected orthogonal partial least squares discriminant analysis S-plot. Note: (**a**) Sti-Pet; (**b**) Ste-Pet; (**c**) Sta-Pet; (**d**) Ro-Pet; (**e**) Lea-Pet; (**f**) Pet-Ste-Lea-Sta-Sti-Ro.

Compared with the principal component analysis (PCA) technique, orthogonal partial least squares discriminant analysis (OPLS-DA) can provide more accurate results when establishing a classification model, thus improving the prediction ability and accuracy of the model. After processing the data based on OPLS-DA, score plots of each group were drawn according to the analysis of the OPLS-DA model, fully demonstrating the metabolite differences among the six parts. In this study, six groups of OPLS-DA S-plot analysis diagrams were established for the metabolites among the six parts for comparison, and significant differences in metabolites among each sample could be seen (Figure 7). The diagnostic metrics for the OPLS-DA model include R^2^X and R^2^Y, representing the explained variance of the X and Y matrices, and Q^2^, denoting predictive accuracy. A high degree of model stability and reliability is indicated when all three parameters approach a value of 1. Among the prediction parameters of the 18-group evaluation model, these three indicators are all close to 1, indicating that the model is reliable, and differential flavonoid metabolites between different parts can be further screened based on the variable importance in projection (VIP) value analysis (Figure 8).

### 3.8. Screening of Differential Metabolites

**Figure 9:**
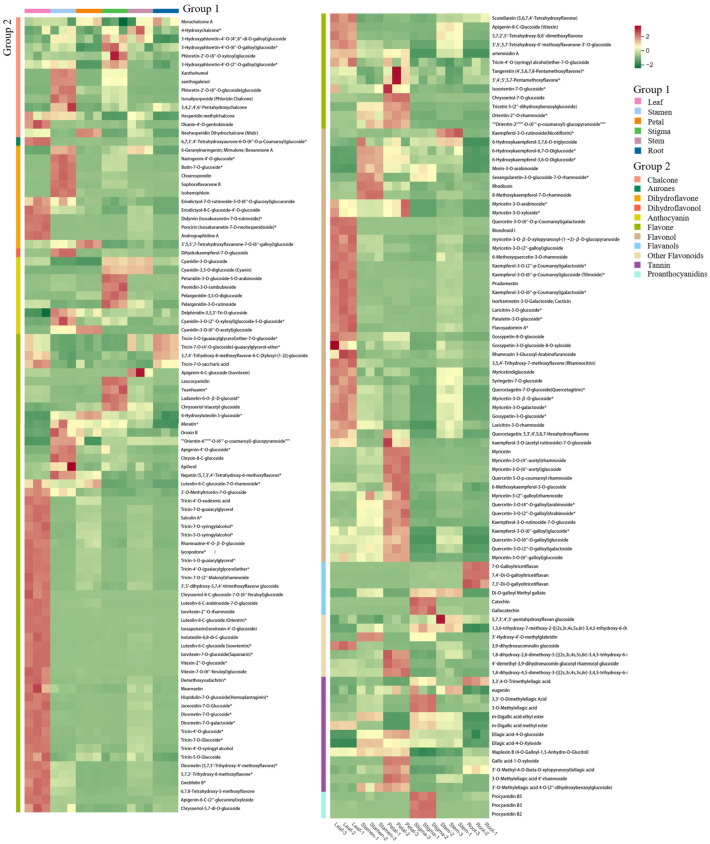
Key differential metabolite clustering heat map. “*” indicates required content.

To explore the differences in metabolites among different parts of *Nymphaea* ‘Blue Bird’, with the criteria of fold change (FC) ≥ 2 or ≤0.5 and VIP value > 1, comparisons were made among the parts of Lea-Pet, Ro-Pet, Ste-Pet, Sta-Pet, and Sti-Pet, so as to analyze the differences in flavonoid metabolites among the six parts (Table 1). Between Ro-Pet, there were 359 metabolites (115 up-regulated substances and 228 down-regulated substances). Among the top 20 metabolites in terms of FC, 13 were down-regulated and 7 were up-regulated. Between Sti-Pet, there were 359 differential metabolites, with 129 metabolites up-regulated and 230 metabolites down-regulated. Among the top 20 metabolites in terms of FC, 14 were down-regulated and 6 were up-regulated. Between Lea-Pet, Ste-Pet, and Sta-Pet, there were 379, 339, and 301 differential metabolites, respectively. The number of up-regulated and down-regulated metabolites was relatively balanced (197 up-regulated and 182 down-regulated; 170 up-regulated and 169 down-regulated; 158 up-regulated and 143 down-regulated), but the number of up-regulated substances was more than that of down-regulated substances, and the main differential metabolites were flavones, flavonols, dihydroflavones, etc. (Figure 9). In addition, among the top 20 metabolites in terms of FC between Sta-Pet, only 1 flavone (apigenin-4′-O-(2′′-O-p-coumaroyl)-β-D-glucoside) was down-regulated, and 19 were up-regulated (tamarixetin-3-O-rhamnoside, etc.). The differential metabolites between Ro-Pet and Sti-Pet were dominated by the down-regulation mode, and flavones and flavonols were the main components of the differential metabolites (myricetin-3-O-(2′′-galloyl-4′′-acetyl) rhamnoside, Nympholide A, myricetin-3-O-(6′′-acetyl) glucoside, pentahydroxyflavone 5-(2′′-dihydroxybenzoyl) glucoside, etc.

**Figure 10:**
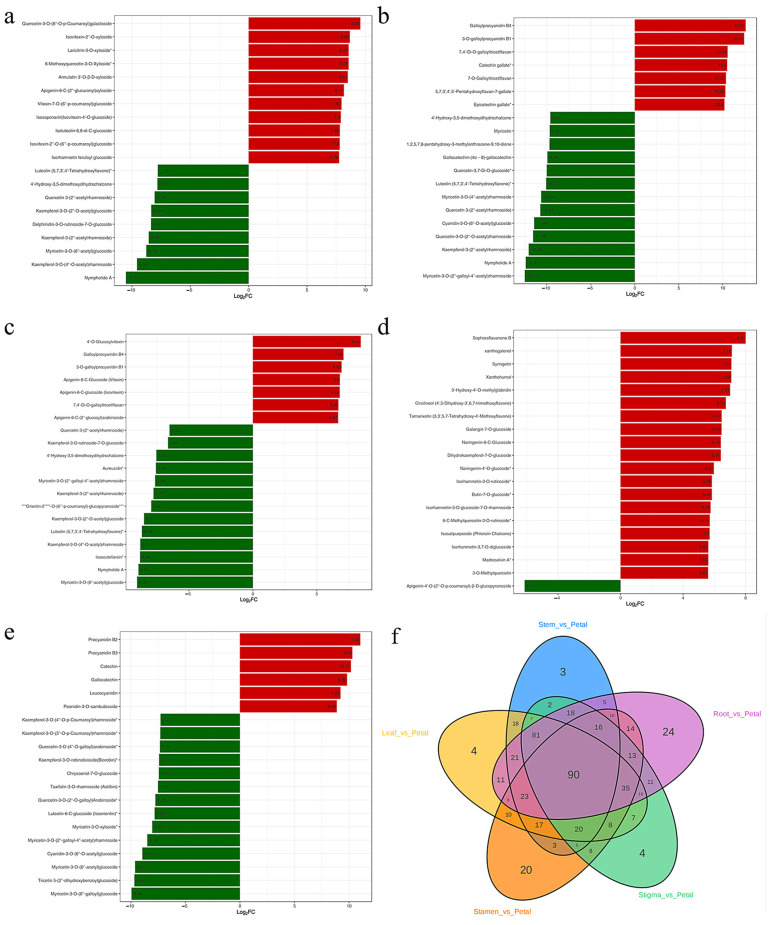
Bar chart of metabolites in the top 20 fold change. Note: (**a**) Lea-Pet; (**b**) Ro-Pet; (**c**) Ste-Pet; (**d**) Sta-Pet; (**e**) Sti-Pet. (**f**) Venn diagram of unique differential metabolites. “*” indicates that there is additional annotation information about this compound that needs to be viewed. Red represents upregulation of metabolite content, while green represents downregulation of metabolite content.

Meanwhile, cluster heatmap analysis was conducted on the screened key differential metabolites. Chalcones had a relatively high content in stamens and stigmas, while the content in the other four parts was not significant. Most flavones and flavonols had a high content in leaves and the lowest content in roots (Figure 10). Among them, flavonoids were rich in variety, mainly tricin, luteolin and their derivative glycosides, which were also the main synthetic substances in the flavone pathway. Compared with other parts, tricin, luteolin and their derivative glycosides in leaves were significantly upregulated. Quercetin, kaempferol and their derivative glycosides, as key nodal substances in the flavone pathway, had a high content in leaves and petals. Compared with other parts, the content of other flavonoids and tannins was significantly upregulated in petals. Among them, tannins such as 1,8-dihydroxy-2,6-dimethoxy-5-[(2s,3r,4s,5s,6r)-3,4,5-trihydroxy-6-(hydroxymethyl)oxy-2-yl]oxy-9-anthrone, 3-(4-hydroxybenzyl)-5,7-dihydroxy-6-methoxy-chroman-4-one glucoside and 3-O-methyl ellagic acid were more abundant in petals. Anthocyanins and proanthocyanidins were significantly upregulated in stigmas, such as pelargonidin-3-O-rutinoside, cyanidin-3-O-glucoside and the isomer of proanthocyanidin B2.

There were 24 unique differential metabolites in Ro-Pet, mainly tannins. In Sta-Pet, there were 20 unique differential metabolites, mainly flavonols, flavones, and other flavonoids. In Sti-Pet, Lea-Pet, and Ste-Pet, there were 4 (such as Euphorbia factor D), 4 (such as Euphorbia factor D), and 3 (such as chrysin) unique differential metabolites, respectively. When comparing the six parts as comparison groups with each other, a total of 90 differential metabolites were identified among different samples (Figure 10f).

### 3.9. KEGG Enrichment Analysis of Differential Metabolites

**Figure 11:**
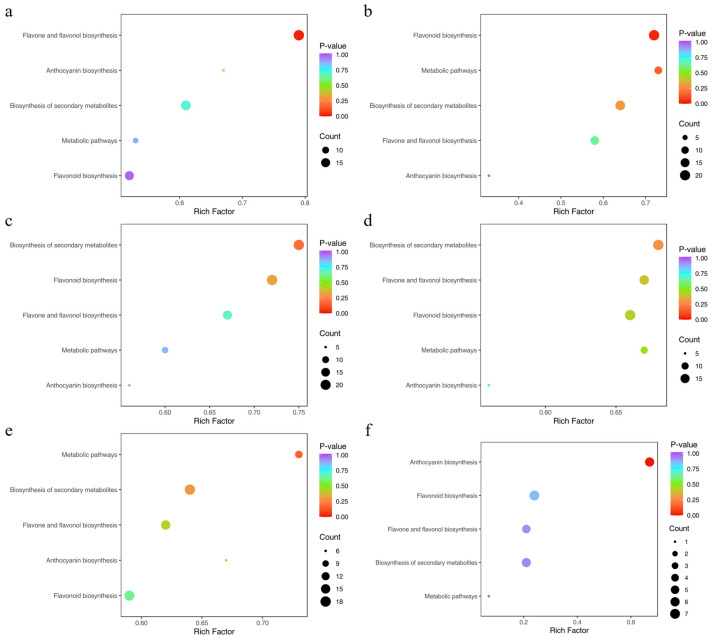
KEGG enrichment of differential metabolites. Note: (**a**) Lea-Pet; (**b**) Ro-Pet; (**c**) Sta-Pet; (**d**) Ste-Pet; (**e**) Sti-Pet; (**f**) Pet-Ste-Lea-Sta-Sti-Ro.

To elucidate the biological relevance of the differential metabolites, we performed KEGG pathway annotation followed by enrichment analysis for all comparison groups, which allowed us to screen for metabolic pathways with significant enrichment. 35, 33, 38, 35, and 33 metabolites were, respectively, annotated to the corresponding pathways of differential metabolites in Lea-Pet, Ro-Pet, Sta-Pet, Ste-Pet, and Sti-Pet. The flavonoid biosynthesis pathway and the secondary metabolite biosynthesis pathway were the pathways involving the most metabolites, with 93 differential metabolites. The flavone and flavonol biosynthesis pathways, the metabolite biosynthesis pathway, and the anthocyanin biosynthesis pathway all involved more than 25 differential metabolites, which could be regarded as the main research pathways for the chemical composition differences in different parts of *Nymphaea* ‘Blue Bird’ (Figure 11). In addition, the differential metabolites of Lea-Pet were mainly enriched in the flavone and flavonol biosynthesis, with 19 metabolites; those of Ro-Pet were mainly enriched in the flavonoid biosynthesis and secondary metabolism pathways, with 21 and 18 differential metabolites, respectively; the differential metabolites of Sta-Pet were mainly enriched in the secondary metabolism pathway and flavonoid biosynthesis, with 21 differential metabolites enriched in both pathways; the differential metabolites of Ste-pet were mainly enriched in the secondary metabolism pathway and flavone and flavonol biosynthesis, with 19 differential metabolites enriched in both pathways; the differential metabolites of Sti-Pet were mainly enriched in the flavonoid biosynthesis pathway and secondary metabolism pathway, with 17 and 18 differential metabolites enriched in the two pathways, respectively.

Based on the KEGG metabolic pathway analysis results of different tissue parts of *Nymphaea* ‘Blue Bird’, a total of 8 main differential metabolites were screened and identified: 3,4,2′,4′,6′-pentahydroxychalcone, pinocembrin, vitexin, luteolin, myricetin, taxifolin (dihydroquercetin), kaempferol-3-O-sophorotrioside, and quercetin.

**Figure 12:**
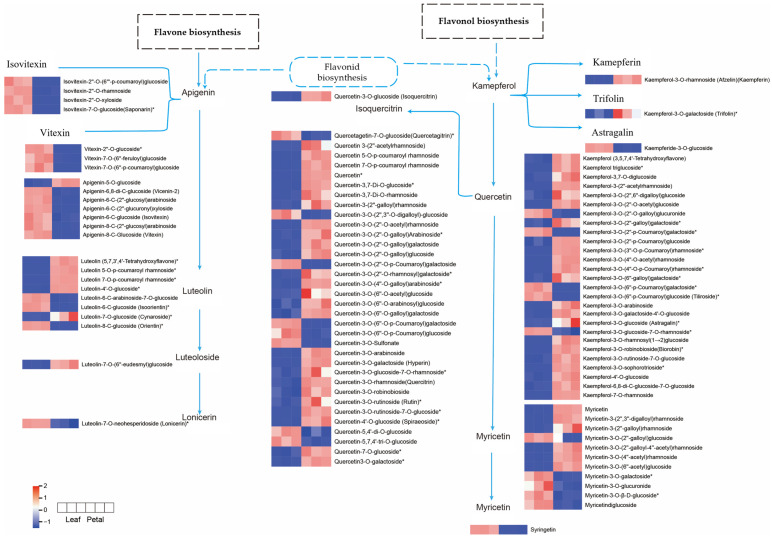
Flavonoid and flavonol biosynthetic pathways of Lea and Pet. Note: The boxes represented metabolites, and the heat map represented accumulation mode of differential metabolites flavonoids, the red color indicated high level of accumulation abundance of flavonoids, the blue color indicated low level of accumulation abundance. “*” indicates that there is additional annotation information about this compound that needs to be viewed.

In the flavone and flavonol biosynthesis pathways enriched in the Lea-Pet comparison group, flavonoid precursor substances such as kaempferol, quercetin, and apigenin, which were annotated, were relatively abundant in Pet. In the accumulation of flavonols, which clearly distinguished the three branch metabolic pathways of kaempferol, quercetin, and myricetin, 3-O-methylquercetin and syringetin were upregulated in Lea, while their upstream kaempferol-type substances, kaempferol-3-O-rhamnoside and kaempferol-3-O-galactoside (trifolin), which were annotated, had a low content in Lea. The content of luteolin, which was annotated for flavones, showed no significant difference between the two parts. However, its upstream products, apigenin-type and vitexin-type substances, were overall downregulated in Lea (Figure 12). Overall, kaempferol-type substances and their downstream metabolite quercetin-type substances were downregulated in Lea, while vitexin-type and apigenin-type substances were upregulated in Lea, and there was no significant difference in the content of their downstream metabolite luteolin-type substances. It is speculated that the conversion of apigenin to downstream products may be the result of the differential expression of CYP75A, but this still needs to be verified by subsequent gene transcriptome research.

**Figure 13:**
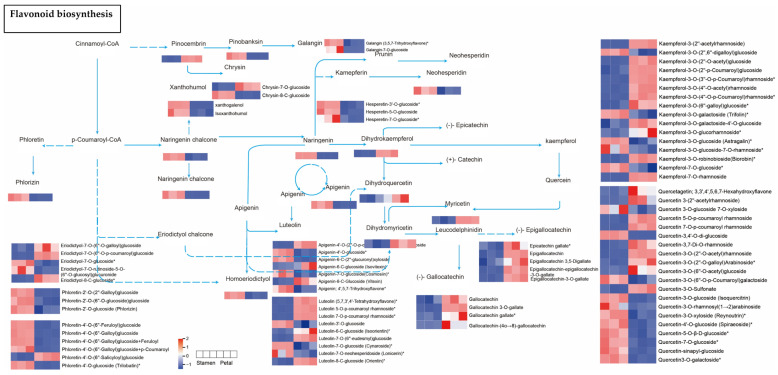
The flavonoid biosynthetic pathway of Sta and Pet. Note: The boxes represented metabolites, and the heat map represented accumulation mode of differential metabolites flavonoids, the red color indicated high level of accumulation abundance of flavonoids, the blue color indicated low level of accumulation abundance. “*” indicates that there is additional annotation information about this compound that needs to be viewed.

In the flavonoid biosynthesis pathway enriched in the Sta-Pet comparison group, the accumulation of flavonoids clearly differentiates among the three branch metabolic pathways of naringenin, pinocembrin, and phloretin. The contents of dihydrokaempferol, dihydroquercetin, dihydromyricetin, and their downstream product (−)-gallocatechin all show a downward trend, while their upstream metabolite naringenin-type substances as a whole show an upward trend. It is speculated that this may be related to the differential expression of F3H and CYP75A and the conversion of kaempferol-type and quercetin-type substances. Phloretin and xanthohumol, which are annotated as flavonoids, are downregulated in Pet, while pinocembrin is upregulated in Pet. They are in different branches of the flavonoid biosynthesis pathway, which may be the reason for the difference in the accumulation of flavonoids between the two parts (Figure 13). In summary, various parts of *Nymphaea* ‘Blue Bird’ contain abundant flavonoids and exhibit different accumulation patterns.

## 4. Discussion

This study successfully optimized the protocols for supercritical CO_2_ fluid extraction and macroporous resin purification of flavonoids from *N.* ‘Blue Bird’. SFE is a recognized green technology that effectively avoids residual organic solvents and has been widely applied to extract active compounds from various plants [11,12]. The SFE parameters determined via Response Surface Methodology in this work were efficient and reliable. The achieved extraction yield (2.56%) was comparable to that reported by Parimala M et al. for flavonoids from water lily leaves (2.81%) [13], yet higher than some studies employing conventional solvent extraction, highlighting the efficiency advantage of SFE for water lily flavonoids. For purification, the polar resin HPD500 was selected from five candidates due to its superior desorption performance (89.25%), aligning with findings by Tungmunnithum et al. during the purification of flavonoids from *Nymphaea lotus*, suggesting a specific affinity of polar resins for water lily flavonoids [14]. The adsorption process was better fitted by the pseudo-second-order kinetic model (R^2^ > 0.99), consistent with Yan Zheng’s conclusions on the adsorption behavior of lotus receptacle flavonoids onto AB-8 resin. This consensus confirms that chemisorption, potentially involving π-π conjugation between flavonoid phenyl rings and the resin matrix, is the rate-limiting step [15,16]. The finalized purification conditions increased the flavonoid purity from 3.05% to 11.46%, representing an approximately 3.76-fold enrichment. This improvement is comparable to the effect achieved by Cao Yan et al. using AB-8 resin to purify raspberry flavonoids (3.3-fold increase) [17], demonstrating the general applicability and high efficiency of our established process. This optimized protocol provides robust technical support for the large-scale preparation and further development of water lily flavonoids.

This study revealed significant tissue-specific variation in the contents of flavonoids and phenolics, along with their associated bioactivities, in *N.* ‘Blue Bird’, with petals identified as the primary site for the accumulation of these bioactive compounds and the resultant biological functions. The strong antioxidant capacity, particularly the potent ABTS^+^ scavenging ability (EC_50_ = 0.15 mg/mL), showed a significant negative correlation with total flavonoid content (R = −0.894). This aligns with the conclusions of Yin et al., who reported a correlation between the antioxidant activity of water lily extracts and their flavonoid content [18]. However, the differential activities observed across various antioxidant assays (DPPH, ABTS, FRAP) suggest that the underlying mechanism involves a synergistic interplay among multiple flavonoid monomers, rather than being attributable to a single component [19]. Regarding antibacterial activity, petal and sepal extracts exhibited strong inhibition against several foodborne pathogens, with greater efficacy observed against Gram-positive bacteria (e.g., *Staphylococcus aureus*). This trend is similar to the antibacterial pattern reported by Lu J et al. for flavonoids from flowers [20]. This selective inhibition against Gram-positive bacteria is often attributed to their relatively simple cell wall structure, contrasting with the permeability barrier posed by the complex double-membrane structure of Gram-negative bacteria [21,22]. Notably, stamen flavonoids demonstrated unique efficacy against Gram-negative bacteria (e.g., *Escherichia coli*), potentially attributable to their distinct flavonoid profile, such as the specific flavonols or dihydroflavonols detected in our metabolomic analysis. Although the overall antibacterial potency of *N.* ‘Blue Bird’ flavonoids was weaker than that of synthetic antibiotic controls, their natural origin and broad-spectrum inhibitory potential position them as promising candidates for developing novel plant-based antimicrobial agents [23]. This work systematically characterizes the bioactivities across different plant parts and provides preliminary correlation with their chemical basis, offering crucial insights for the targeted development of *N.* ‘Blue Bird’ as a source of natural antioxidants and antimicrobials.

In plants, flavonoids are widely present in tissues such as flowers, leaves, and roots. As secondary metabolites, they have various biological functions, including antioxidant properties, regulation of defense responses, and protection against ultraviolet radiation [24]. Multiple studies have shown that the flavonoids in different parts of plants also vary. For example, the contents of flavonoid components in different parts such as the roots, stems, and leaves of Portulaca oleracea are different, and the expression levels of its synthetic metabolic enzymes in different parts also show significant differences [25]. The metabolite distributions in the roots, stems, leaves, and flower tissues of Inula japonica are different, and their pharmacological effects also vary. In this study, the types and differences in metabolites in 9 samples from 6 different tissue parts of *Nymphaea* ‘Blue Bird’ were analyzed. A total of 560 metabolites were identified, with flavones and flavonols being dominant. There were 178 flavonols, 148 flavones, 45 dihydroflavones, and 43 chalcones, indicating that *Nymphaea* ‘Blue Bird’ is rich in metabolites and flavonoids. A total of 85 flavonoid metabolites were identified in Kadsura coccinea, among which flavones and flavonols were the main metabolites. The petals and stamens of Camellia oleifera are also the main sites for the accumulation of flavones and flavonols, which is consistent with this study [26,27]. The Sta of the *N.* ‘Blue Bird’ contain high levels of dihydroflavonols (such as taxifolin). Due to their enhanced hydrophobicity, these compounds may more readily disrupt the outer membrane structure of Gram-negative bacteria, thereby enhancing antibacterial activity. The petals are rich in various quercetin derivatives. This highly aligns with the petal extract demonstrating the strongest ABTS^+^ and DPPH free radical scavenging capacity. It is known that quercetin and its glycosides possess excellent electron-donating ability due to their catechol structure and hydroxyl substitution pattern and are recognized as potent antioxidants [27]. Therefore, the high content of quercetin derivatives in the petals serves as one of the key material foundations for their remarkable antioxidant activity. The contents of flavonoid metabolites such as quercetin-3-O-rhamnoside (quercitrin), kaempferol-3-O-(2″-O-acetyl) rhamnoside, 6-methoxyquercetin-3-O-xyloside, laricitrin-3-O-xyloside, and choerospondin in Pet, Lea, and Sta are relatively high. Evidently, these three parts contain a large amount and diverse types of flavonoids, suggesting that these three parts are the key parts for the effective extraction of flavonoids from *Nymphaea* ‘Blue Bird’. Among them, quercitrin has activities such as anti-inflammation, analgesia, and antidiarrhea, indicating that there is great potential for the development and utilization of flavonoids from *Nymphaea* ‘Blue Bird’ [28]. By combining principal component analysis and cluster analysis to identify the differential metabolites in different parts of *Nymphaea* ‘Blue Bird’, it can promote the full and rational utilization of *Nymphaea* ‘Blue Bird’ resources and lay a scientific foundation for the study of extracting effective chemical components from *Nymphaea* ‘Blue Bird’.

At present, 455 metabolic components in *Nymphaea* ‘Blue Bird’ have been reported, with the flavonoid biosynthesis pathway showing the highest enrichment degree, and flavonoids being the main components [8]. KEGG enrichment analysis revealed that the differential metabolites in *Nymphaea* ‘Blue Bird’ were primarily associated with pathways central to secondary metabolism. The most significantly enriched pathways included the general flavonoid biosynthesis, as well as more specific routes such as flavone and flavonol biosynthesis, and anthocyanin biosynthesis. The significantly differential metabolic components in each part of *Nymphaea* ‘Blue Bird’ are enriched mainly in the flavonoid biosynthesis pathway and metabolic pathways. This helps to further understand the biosynthetic pathways of secondary metabolites in *Nymphaea* ‘Blue Bird’ plants. For example, the significant decrease in the content of pinocembrin in Pet may be due to its synthesis of pinosylvin under the action of F3H in the flavonoid metabolic pathway, resulting in a significant up-regulation of pinosylvin [29]. The contents of flavonoid metabolites in Pet, Sta, and Lea are significantly higher than those in the other three tissue parts. This may be related to the different gene expressions in each tissue part and the different regulations of related genes and the roles played by key enzymes in the biosynthetic pathways, leading to different contents of differential metabolites. Future transcriptomic studies focusing on these key enzymes would be invaluable to validate these hypotheses and elucidate the regulatory network controlling tissue-specific flavonoid biosynthesis in *N*. ‘Blue Bird’. In summary, this study analyzed the differential flavonoid metabolites in different parts of *Nymphaea* ‘Blue Bird’, screened out abundant differential metabolites, and conducted metabolic pathway enrichment analysis of the differential metabolites. However, further in-depth research is still needed on the related metabolic pathways and regulatory genes.

## 5. Conclusions

This study systematically evaluated the potential of *Nymphaea* ‘Blue Bird’ as a source of bioactive flavonoids. We established an efficient and green supercritical CO_2_ fluid extraction process optimized via response surface methodology for flavonoid extraction. Subsequently, purification using HPD500 macroporous resin significantly enhanced flavonoid purity by 3.76-fold. Comprehensive analysis identified petals as the primary accumulation site for flavonoids and phenolics, exhibiting the most potent antioxidant capacity and broad-spectrum antibacterial activity, particularly against Gram-positive bacteria. Employing a widely targeted metabolomics approach, we comprehensively characterized the flavonoid metabolome across different tissues for the first time, identifying 560 metabolites. Furthermore, the study revealed flavonoid biosynthesis as a key metabolic pathway and pinpointed luteolin, myricetin, taxifolin, and quercetin as core differential metabolites strongly associated with the superior bioactivities. In conclusion, this work not only provides an optimized strategy for the sustainable preparation of flavonoids from *N.* ‘Blue Bird’ but also elucidates the metabolic basis for its biological activities at the molecular level, offering a theoretical foundation and data support for its future development and application in functional foods and pharmaceuticals.

## Figures and Tables

**Table 1:** Differential expression of flavonoid metabolites in various parts.

Type	Lea-Pet		Ro-Pet		Ste-Pet		Sta-Pet		Sti-Pet	
	Up	Down	Up	Down	Up	Down	Up	Down	Up	Down
Flavonol	54	77	10	123	52	64	54	36	12	104
Flavone	81	32	14	83	54	37	33	50	17	57
Dihydroflavone	11	17	6	28	10	13	19	10	9	15
Chalcone	9	17	6	21	4	21	20	7	16	11
Other flavonoids	5	13	2	18	9	10	9	10	5	16
Flavanols	1	6	20	4	17	3	1	14	14	6
Anthocyanin	8	6	4	7	9	6	6	5	11	8
Dihydroflavonol	1	5	1	8	1	6	2	5	2	5
Proanthocyanidins	4	0	7	0	1	1	0	1	6	0
Orange ketone	2	3	0	5	2	3	0	1	0	0
Tannin	21	6	8	24	11	5	14	4	23	6
Total	197	182	78	321	170	169	158	143	115	228

## Data Availability

The original contributions presented in this study are included in the article/Supplementary Material. Further inquiries can be directed to the corresponding authors.

## Supplementary Materials

The following supporting information can be downloaded at https://www.mdpi.com/article/10.3390/life15121895/s1, Supplementary Methods: Detailed Procedures for Flavonoid Extraction and Purification; Figure S1: Single factor affecting the purification effect of macroporous resin; Table S1: Sample numbers of each tissue part of *Nymphaea* ‘Blue Bird’; Table S2: Box–Behnken experimental design and results. References [30,31] are cited in the supplementary materials.

## Abbreviations

The following abbreviations are used in this manuscript:

SFE	Supercritical CO_2_ Fluid Extraction
ABTS	2,2′-Azinobis(3-ethylbenzothiazoline-6-sulfonic acid) diammonium salt
DPPH	2,2-Diphenyl-1-picrylhydrazyl
MIC	Minimum Inhibitory Concentration
MBC	Minimum Bactericidal Concentration
LB	Luria–Bertani
OPLS-DA	Orthogonal Partial Least Squares Discriminant Analysis
FC	Fold Change
FRAP	Ferric Reducing Antioxidant Power
DP	Declustering Potential
CE	Collision Energy
HCA	Hierarchical Cluster Analysis
PCA	Principal Component Analysis
VIP	Variable Importance in Projection
