# Supplementary material for: Flavonoid Composition and Bioactivities of Nymphaea ‘Blue Bird’: Analysis, Purification, and Evaluation
Source: Life (Basel). 2025 Dec 11;15(12):1895. doi: 10.3390/life15121895 (PMC12734380; doi:10.3390/life15121895)
Supplement: Supplementary file 1 [file life-15-01895-s001.zip › Supplementary Methods.pdf]

Supplementary Methods: Detailed Procedures for Flavonoid Extraction and Purification

1. Overview of Analytical and Purification Methods

An appropriate amount of anhydrous ethanol was used to dissolve 20 mg of the rutin standard, and the volume was made up to 10 mL to prepare a 2 mg/mL standard stock solution. Then, 1 mL aliquots of standard working solutions with concentrations of 0.2, 0.4, 0.6, 0.8, and 0.85 mg/mL were transferred into separate 5 mL centrifuge tubes. To each tube, 150 μL of a 5% $NaNO_2$ solution was added. The mixtures were vortexed thoroughly and allowed to react for 6 minutes. After complete reaction, 150 μL of a 10% $Al(NO_3)_3$ solution was added, followed by another 6-minute reaction period. Finally, 2 mL of a 4% NaOH solution was added, and the volume in each tube was brought up to the mark with ethanol. The solutions were left to stand for 15 minutes. The absorbance of each concentration gradient was measured at 510 nm, and a standard curve was plotted [30]. The extraction yield of flavonoids was calculated according to Formula (S1). Using the standard solution concentration (C) and absorbance (A), the standard curve was plotted, yielding the standard curve equation: A = 3.650C - 0.1004 (R^2 = 0.9895).

$$\text{Flavonoid Yield (\%)} = (C \times V \times N) / M \times 100\% \quad \text{(S1)}$$

Where:
C: Concentration of flavonoids (mg/mL)
V: Volume of the extract or the volume after dilution (mL)
N: Dilution factor
M: Mass of the dried sample powder (mg)

2. Single-Factor Experiments and Box-Behnken Response Surface Methodology Design

Using the petals of the water lily (*Nymphaea* 'Blue Bird') and taking the total flavonoid extraction yield as the evaluation index, single-factor experiments were conducted to investigate the effects of extraction pressure (10, 15, 20, 25, 30 MPa), extraction temperature (30, 35, 40, 45, 50°C), extraction time (60, 80, 100, 120, 140 min), and flow rate (4, 8, 12, 16, 20 L/min) on the flavonoid extraction yield. Based on the results of the single-factor experiments, a response surface methodology experiment was designed.

3. Macroporous Resin Screening

3.1. Adsorption and Desorption of Macroporous Resins

The blotted-dry macroporous resin (0.5 g) was mixed with 20 mL of the crude flavonoid extract (concentration: 0.30–0.44 mg/mL) in a 50 mL centrifuge tube. The tube was then shaken (150 rpm) at 25°C for 24 h in a horizontal shaker. After adsorption, the resin was washed 2–3 times with distilled water, transferred back into a 50 mL centrifuge tube, and mixed with 20 mL of 95% ethanol. The tube was shaken again (150 rpm) for 24 h. Finally, the flavonoid concentration in the desorption solution was measured. The adsorption and desorption ratios were calculated using Formulas (S2) and (S3), respectively.

$$A(\%) = \frac{(C_0 - C_t) \times V}{C_0} \times 100\% \quad \text{(S2)}$$

Where:
A: Adsorption ratio (%)
C_0: Initial flavonoid concentration (mg/mL)
C_e: Flavonoid concentration after adsorption (mg/mL)

$$A(\%) = \frac{C - V}{(C_0 - C_t) \times V_0} \times 100\% \quad \text{(S3)}$$

Where:
A: Desorption ratio (%)
C: Flavonoid concentration in the desorption solution (mg/mL)
V: Volume of the desorption solution (mL)
V_0: Volume of the initial flavonoid solution (mL)
C_0: Initial flavonoid concentration (mg/mL)
C_t: Flavonoid concentration after adsorption (mg/mL)

3.2. Adsorption Kinetics of Two Resins on Flavonoids from Nymphaea 'Blue Bird'

Two initially screened resins, NKA-9 and HPD500 (1.0 g each), were placed in separate 50 mL centrifuge tubes. Then, 20 mL of the crude flavonoid extract was added to each tube. The tubes were shaken at 25°C and 200 rpm for 8 hours, and the flavonoid concentration in the adsorption solution was measured at different time intervals. The adsorption kinetics were analyzed using three kinetic models: the pseudo-first-order (S4), pseudo-second-order (S5), and Kanan intra-particle diffusion models (S6) [31].

Pseudo-First-Order Kinetic Model:

$$\ln(Q_e - Q_t) = -K_1 t + \ln Q_e \quad \text{(S4)}$$

Pseudo-Second-Order Kinetic Model:

$$\frac{l}{Q_t} = \frac{1}{k_2} \times \frac{1}{t} + \frac{1}{Q_2} \quad \text{(S5)}$$

Kannan Intra-Particle Diffusion Model:

$$Q_t = k_d \times t^{\frac{1}{2}} + C \quad \text{(S6)}$$

Where:
Q_e: Adsorption capacity at equilibrium (mg/g)
Q_t: Adsorption capacity at time *t* (mg/g)
t: Adsorption time (h)
K_1: Pseudo-first-order rate constant
K_2: Pseudo-second-order rate constant
K_d: Intra-particle diffusion rate constant
C: Equation constant

4. Single-Factor Experiments Investigating the Effects on Macroporous Resin Adsorption and Desorption

4.1. Selection of Loading Volume

A total of 0.5 g of pre-treated macroporous resin was weighed and mixed with different volumes (5, 10, 15, 20, 25 mL) of a water lily flavonoid solution of known concentration in separate containers. The mixtures were then shaken in an incubator shaker at 25°C and 200 rpm for 24 hours, after which the adsorption ratio was determined.

4.2. Selection of Sample Concentration

The water lily flavonoid extract was prepared into sample solutions at different concentrations. Then, 0.5 g of resin was weighed into centrifuge tubes, and 10 mL of the flavonoid extract at varying concentrations (0.147, 0.195, 0.269, 0.359, 0.418, 0.474, 0.616 mg/mL) was added to each tube. After shaking at a constant temperature for 24 hours, the concentration of the solution was measured, and the adsorption ratio was calculated.

4.3. Selection of Sample Solution pH

The pH of the flavonoid extract was adjusted using hydrochloric acid and sodium hydroxide solutions. Then, 0.5 g of pre-treated macroporous resin was weighed into conical flasks, and 10 mL of the flavonoid extract at different pH levels (3.0, 5.0, 7.0, 9.0, 11.0) was added to each flask. After shaking at a constant temperature for 24 hours, the concentration of the solution was measured, and the adsorption ratio was calculated.

4.4. Selection of Eluent Concentration

A total of 0.5 g of prepared macroporous resin was weighed into conical flasks, and a known concentration of water lily flavonoid solution was added for adsorption. Subsequently, 10 mL of ethanol solutions at different concentrations (50%, 60%, 70%, 80%, 90%) were added respectively for oscillating desorption over 24 hours. The desorption ratio was then determined.

4.5. Selection of Eluent Volume

A total of 5 g of prepared macroporous resin was weighed and packed into a column using the wet packing method, with the elution flow rate controlled at 1 mL/min. Different volumes (10, 20, 30, 40, 50 mL) of a 90% ethanol solution were used for elution. The eluate concentration was measured, and the desorption ratio was determined.

4.6. Selection of Elution Flow Rate

A total of 5 g of prepared macroporous resin was weighed and packed into a column using the wet packing method. A 90% ethanol solution was used as the eluent, with the flow rate controlled at different levels (0.5, 1, 1.5, 2, 2.5, 3 mL/min) for elution. The eluate concentration was measured after elution, and the desorption ratio was determined.
