# Supplementary figures and images for: Flavonoid Composition and Bioactivities of Nymphaea ‘Blue Bird’: Analysis, Purification, and Evaluation
Source: Life (Basel). 2025 Dec 11;15(12):1895. doi: 10.3390/life15121895 (PMC12734380; doi:10.3390/life15121895)

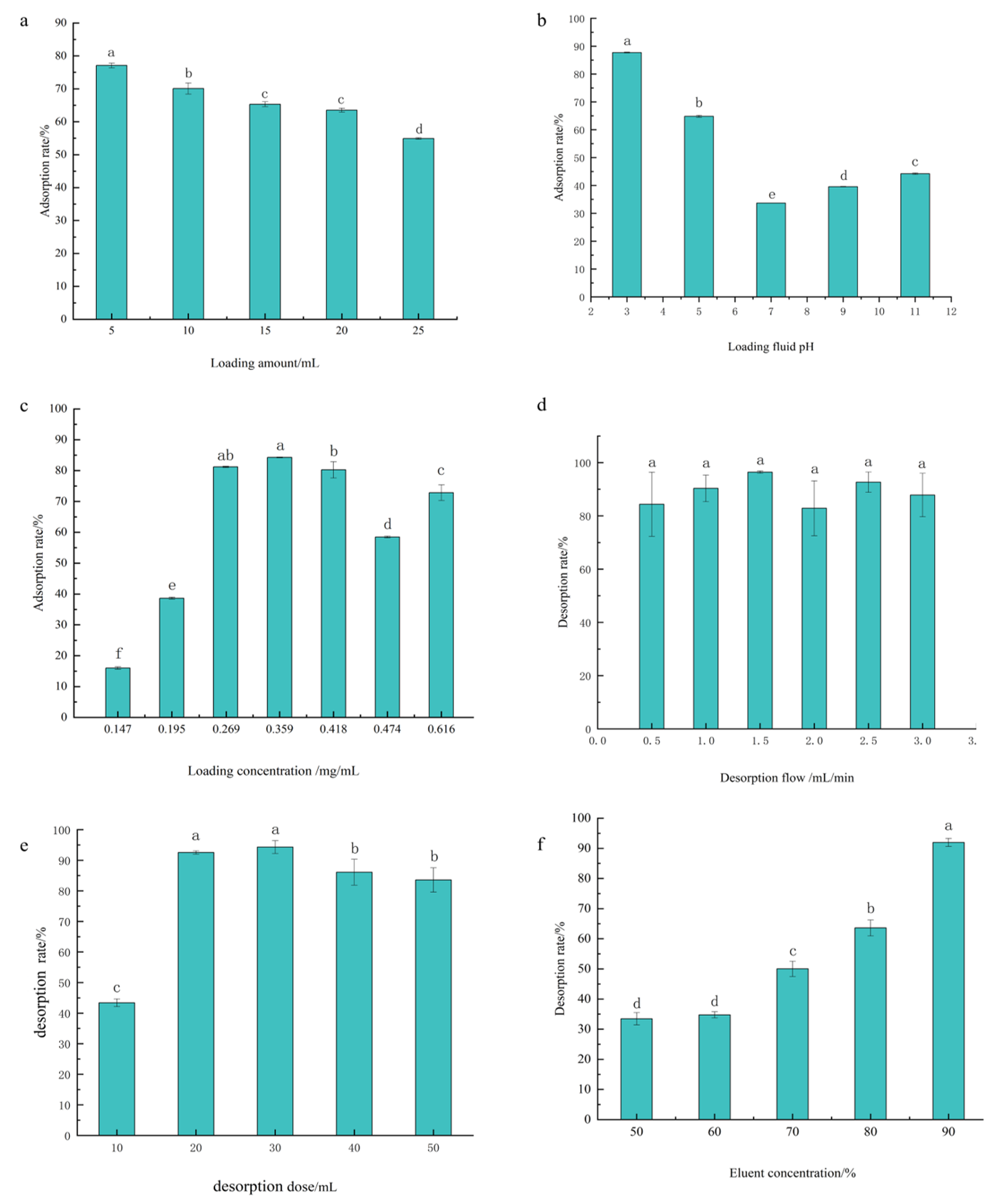

Supplement: Supplementary file 1 [file life-15-01895-s001.zip › Supplementary Figure S1.png]
